# Post-stroke Movement Disorders: Clinical Manifestations and Pharmacological Management

**DOI:** 10.2174/157015912803217341

**Published:** 2012-09

**Authors:** Antonio Siniscalchi, Luca Gallelli, Angelo Labate, Giovanni Malferrari, Caterina Palleria, Giovambattista De Sarro

**Affiliations:** 1 Department of Neuroscience, Neurology Division, “Annunziata” Hospital, Cosenza, Italy; 2 Chair of Pharmacology, Department of Health Science, School of Medicine, University of Catanzaro, Clinical Pharmacology Unit, Mater Domini University Hospital, Catanzaro, Italy; 3 Institute of Neurology, University of Catanzaro, Catanzaro, Italy; 4 Department of Neurology, Santa Maria Nuova Hospital, Reggio Emilia, Italy

**Keywords:** Post-stroke, movement disorders, hyperkinetic movements disorders, hypokinetic movements disorders, GABA, Dopamine, post-stroke recovery.

## Abstract

Involuntary abnormal movements have been reported after ischaemic and haemorrhagic stroke. Post stroke movement disorders can appear as acute or delayed sequel. At the moment, for many of these disorders the knowledge of pharmacological treatment is still inadequate. Dopaminergic and GABAergic systems may be mainly involved in post-stroke movement disorders. This article provides a review on drugs commonly used in post-stroke movement disorders, given that some post-stroke movement disorders have shown a partial benefit with pharmacological approach.

## INTRODUCTION 

Movement disorders represent a documented complication of stroke; both hyperkinetic and hypokinetic movement disorders have been explained after ischaemic and haemorrhagic stroke [1,2]. Hemichorea-hemiballism [2,6-10], dystonia [2-7], tremor [2,7,11-13], myoclonus [1,2,7,9], parkinsonism [2,6,9,14] have been reported after stroke [2,9,10] as well as in a delayed setting [2,7,10] or as progressive conditions [2,7]. In contrast, transient dyskinesias could represent a symptom of transient ischaemic attacks [1,2,15].

The frequency of post-stroke abnormal movements is unclear. One study which included 1,500 stroke patients, showed that 3.7% of these patients developed movement disorders [9], while Ghika-Schmid *et al*. [10], described a prevalence of movement disorders in 1% of post stroke patients and an incidence of 0.08%. In both studies hemichorea was the most common movement disorder [9,10].

The time course for the development of movement disorders varies considerably from the day of the onset of stroke to several years later and it also depends on the type of movement disorder [2,7,9,10]. In fact, chorea appears earlier (mean 4.3 days post-stroke) while parkinsonism much later (mean 117.5 days post-stroke) [9]. However, there is a wide variability within each movement disorder; the delay in developing dystonia after stroke can be from 1 day to 5 years [2] and this may affect the time required for the partial recovery of motor functions and the development of pathological circuitry [7].

This review aims to delineate the efficacy and safety of drugs used in the treatment of post-stroke movement disorders. 

PubMed and Medscape were reviewed (1990–2011) and English-language articles were identified with the key words: movement disorders, post stroke and drug treatment. Secondary searches included articles cited in sources identified by the primary search.

## PATHOPHYSIOLOGY OF MOVEMENTS DISORDERS

**Fig. (1):**
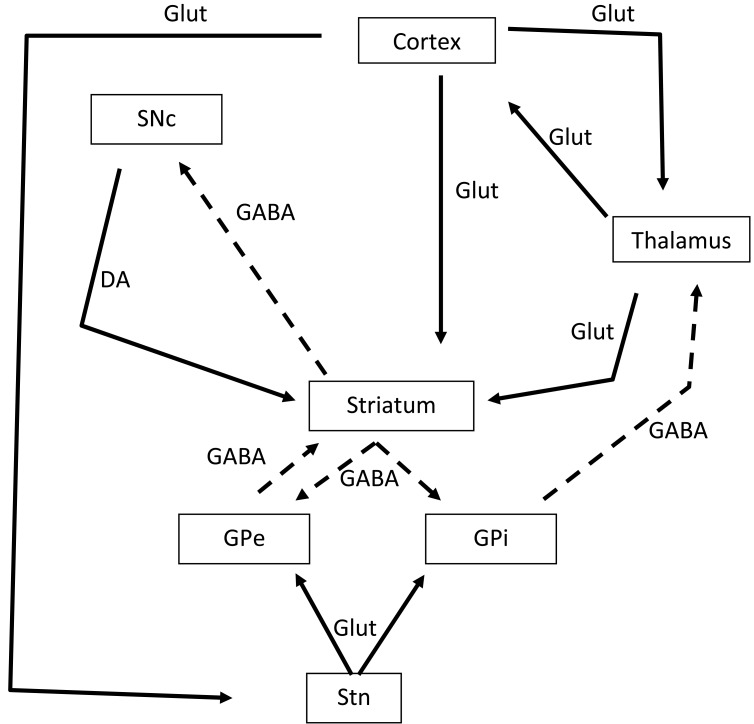
**Representation of neuronal connections in the basal ganglia.** Broken lines represent inhibitory connections, and unbroken lines represent stimulatory connections. SNc: substantial nigra pars compacta; GPi: globus pallidus pars interna; STn: subthalamus nucleus; SNr: substantial nigra pars reticulate; GPe: globus pallidus pars externa. DA: dopamine; Glut: glutamate; GABA: *gamma-aminobutyric acid A*.

The basal ganglia represents an area in the brain most often interested in post-stroke movement disorders (Fig. **1**). Basic functions of the pathways of the motor cortex is to act as a cortical feedback loop in which signals from the neo-cortex are relayed through the striatum, pallidum and thalamus back to the cortex. The cortex sends excitatory inputs to the striatum; the striatum inhibits the pallidum which, in turn, inhibits the thalamus [16]. The effect of cortical activity is to trigger the striatum to release the thalamus from pallidum inhibition, thus allowing thalamic outputs to excite the cortex [16]. These direct pathways are modulated by excitatory indirect pathways from both substantia nigra (dopaminergic) and subthalamic nucleus (glutamate). At rest, neurones in the striatum are quiescent and those in pallidum are active, thereby inhibiting the thalamic excitation of motor cortex. Before and during a movement, the striatum becomes active and inhibits the pallidum, allowing more excitation of the motor thalamic nuclei and cortex [16]. Both small vessel diseases and small deep infarcts represent the most common sub-types of stroke leading to abnormal movements [9,10]. In Alarcon’s study, patients with deep lesions in the basal ganglia, thalamus and brain stem, whom developed abnormal movements, have significantly more haemorrhages compared to patients with infarct lesions [9]. Large and medium vessel atherothrombosis and cardiac embolisms are other potential causes of strokes leading to abnormal movements [7,9].

Pathophysiologically, these disorders are characterized by a lower basal ganglia output resulting in a disinhibition of the thalamo-cortical systems, releasing cortical motor areas and allowing movements that are normally suppressed. In patients with vascular parkinsonism, these symptoms could be caused by vascular lesions disrupting the interconnecting fibre tracts between basal ganglia, thalamus and motor cortex which leads to disruption of both the sensorimotor integration [9] and the descending reticular pathways to the major centres of the brain stem. Furthermore, the specific manifestations of movement disorders may be determined by the presence or absence of other abnormalities, such as the level of basal ganglia output synchronization [17]. Recently Lin *et al*. [18], showed that an impairment of the GABAergic system in substantia nigra may induce involuntary movement disorders. Moreover, a degeneration of dopaminergic nerve terminals after cerebral ischemia was described in the strionigral system [19].

Dopaminergic and GABAergic systems may be mainly involved in post-stroke movement disorders. In particular, GABA mediates the majority of fast inhibitory synaptic transmission in the CNS; glycine is the inhibitory transmitter for some neurons in the brainstem and spinal cord [20]. GABA and glycine receptors are pentameric arrangements of subunits around a central pore that conducts chloride when opened by transmitter binding, thus inhibiting the postsynaptic cells. Therefore, drugs that are capable to modulate Dopaminergic or GABAergic tone are usually used in the management of post-stroke movement disorders (Table **1**).

## DRUG TREATMENT

The drugs used in each condition are shown in Table **1**. However, no firm guidelines for the management of movements disorders are available at the moment due to the limited number of patients. 

## HEMICHOREA-HEMIBALLISM, DYSTONIA

Chorea consists in irregular unilateral, purposeless, abrupt, rapid, brief, jerky, un-sustained movements that may involve all parts of the body, but predominantly interests distal parts and most often only one side of the body (hemichorea) [21-23]. Ballism, a severe form of chorea, is characterized by wide amplitude, flinging movements, usually involving the proximal limbs and most often affects only one side of the body (hemiballism) [24-26]*.* Usually a lesion in the contralateral subthalamic nucleus is the cause of ballism, though**,** pathology in other subcortical areas may also be involved [21,22,24]. 

Hemichorea post-stroke may be induced by both ischemic and haemorrhagic stroke [27]. Nevertheless, chorea is an unusual complication of acute vascular lesions, observed in less than 1% of patients with acute stroke [28]. Rarely, vascular ballism and chorea can occur bilaterally [21,22,24-26]. Many patients with ballism have distal choreic movements and, when recovery takes place, hemiballism often changes to hemichorea and hemidystonia [21,22]. 

Alarcon *et al*. [9], showed that in eight patients with hemiballismus, one had subthalamic lesions, one pallidal lesion, and six thalamic lesions. 

Lesions of the subthalamic nucleus presumably reduce the inhibitory output of globus pallidus in the thalamus, thus increasing the excitatory drive to the cortex with the development of contralateral hyperkinetic movements [29,30]. The different topography of each lesion in patients with hemichorea showed the importance of cortical-basal network in the development of movement disorders. The moyamoya disease is an uncommon cause of vascular chorea, it is an intracranial vasculopathy that evidences an ischemic lesion or, less commonly, hemorrhagic stroke of the basal ganglia [31]. 

Post-stroke dyskinesia is usually reported as pure movement disorders, but it can be variable, enclosing several components and be difficult to classify [10,32]. 

Since chorea and hemiballismus may be part of the same spectrum of diseases, the term “hemichorea–hemiballism” is used. [1]. Athetosis and sometimes dystonia may also be combined with hemichorea–hemiballism [1].

Pharmacological treatment includes anti-dopaminergic drugs including typical and atypical neuroleptics and catecholamine-depleting agents. Typical neuroleptic agents (i.e. haloperidol, pimozide, perphenazine and fluphenazine) block dopamine 1 (D1) and D2 receptors [33] and represent first line treatment of hemichorea-hemiballismus [2,34,35]. In particular, Ristic *et al* [35], studied, for a mean period of 30 months, a cohort 27 consecutive patients with hemiballismus as a first manifestation of ischemic strokes treated with haloperidol (<10 mg/day) or a combination of haloperidol and diazepam (<10 mg/day). The authors documented no response to treatment in 2 patients (7%), a complete improvement of symptoms in 15 patients (56%), and a residual choreic movement in 10 patients (37%). Atypical neuroleptics (i.e. olanzapine, quetiapine and sulpiride) are D3 and D4 receptor antagonists [33] and their use is related to a low risk of parkinsonism and delayed dyskinesia [2,36]. Moreover no increased risk of cerebrovascular events had been documented during atypical neuroleptic treatment (e.g risperidone or olanzapine) [37]. In experimental animal models of cerebral ischemia the olanzapine, risperidone, and quietapine showed evidence for neuroprotection after permanent focal cerebral ischemia [38-40]. 

Clozapine had been successful in refractory cases because it induced agranulocytosis in 1% of treated patients [2]. Both tetrabenazine - which depletes presynaptic dopamine and blocks post-synaptic dopamine receptors - and reserpine - that depletes presynaptic stores of catecholamines and serotonin - are effective in hemiballismus treatment even if these drugs can induce depression, hypotension and parkinsonism [41]. Either clonazepam which is a long acting central-type GABA-A receptor agonist [42], or valproic acid which is an indirect agonist of GABA receptors, can be used in both chorea and hemiballismus, in adults as well as in children [21,41,43,44]. 

In particular Pena *et al*., [43] evaluated the efficacy of haloperidol (3 mg/day, b. i. d.), carbamazepine (15-20 mg/kg/day, b.i.d.), and valproic acid (20 mg/kg/day, b.i.d.) in the treatment of Sydenham´s chorea in 18 people (aged 7-15 years) and documented that valproic acid is safe and effective and nonetheless induces a rapid response.

Levetiracetam and topiramate have shown several action mechanisms (i.e. inhibition of calcium release from intraneuronal stores, modulation of GABA- and glycin-gated currents) and have been reported to be useful in the management of patients with hemichorea/hemiballism [45], vascular hemichorea/hemiballism [46,47] and vascular generalized chorea [48].

D’Amelio *et al*., [45] reported a 78 year old man with hemichorea who remarkably improved after treatment with levetiracetam (final dosage 500 mg/day) however, the authors failed to document the long time effects of levetiracetm because the drug was dismissed due to the presence of skin rash.

Dystonia is a neurological disorder dominated by sustained muscle contractions, which frequently causes twisting, repetitive, and patterned movements or abnormal postures. Dystonic movements can be slow, manifested by prolonged dystonic spasms resulting in abnormal postures, or can be rapid and jerky-like movements [21,25,49].

Dystonia can involve the ipsilateral side of the face, arm and leg (hemidystonia) or can be segmental or focal [3,7,9]. Focal dystonia can affect the hand (most commonly), foot, facial or lingual muscles [7,9]*.* Dystonia can be associated with abnormal movements such as jerks (dystonic myoclonus), tremor like (dystonic tremor) or athetoid movements [1,5]. The abnormal twisting movements of dystonia are caused by co-contraction of agonist and antagonist muscles and voluntary movements worsen this condition [1]*.* Vascular dystonia is the most common cause of secondary movement disorders [50]. In contrast to post-stroke spasticity, which is associated to muscle weakness and increased by inhibitory impulses, dystonia is associated to muscle overactivity caused by reduced inhibition at many levels of the motor system [2]. In patients with vascular dystonia, the lesions were most frequently seen in the lentiform nucleus, particularly when involving the putamen [6,51,52]. In Alarcon study, dystonia of the hand was described as secondary to a pontine haemorrhage in a patient, while foot dystonia with a severe proprioceptive deficit in two patients [9]. In all patients, dystonia worsened while they walked up or down hills; this effect could be related to proprioceptive dysfunction [7]. Russmann *et al*. [53], reported that dystonia represents a rare sequela of lenticular infarction. Botulinium toxin, blocking the release of acetylcholine has been used in the management of dystonia [2,25]. Other treatments include benzodiazepines, baclofen, anticholinergic drugs and dopamine-depleting/blocking agents [3]. Clonazepam and diazepam are used in the treatment of focal, segmental and generalised dystonias. Higher dosages are limited because may cause drowsiness. Like botulinium toxin, the anti-cholinergic drugs block the release of acetylcholine, deactivating muscle contractions. Trihexyphenidyl [benzhexol] is the most common drug among this class, but it may be more useful in younger patients because it induces confusion and constipation in the elderly [2,3]. Tetrabenazine can also be helpful, even if it may paradoxically induce dystonia. Combining benzhexol and tetrabenazine can be very effective in younger patients [54]. Attacks of kinesigenic paroxysmal dystonia can be controlled with other antiepileptic drugs [21,25,55].

In particular, Richter and Loscher [56] documented that gabapentin at lower dosages (5-10 mg/Kg/day) can improve the severity of dystonia in an experimental animal model of idiopathic paroxysmal dystonic choreoathetosis. In contrast, the administration of higher doses of gabapentin (10-20 mg/kg/day) may rather bear the risk to worsen dystonia.

The non- kinesigenic form of paroxysmal dystonia are less responsive to pharmacological treatment, even if clonazepam can be used [57].

## TREMOR

Poststroke tremor occurs most commonly with a multifocal or segmental distribution and is related to the action, even if some exhibit a mixture of postural and kinetic components [7,9,12,58]. The term ‘rubral’ or midbrain tremor was used to describe a resting tremor that becomes more severe on posture maintenance and most severe at intention [12]. Tremor can be caused by strokes or underlying CNS vascular malformations. Acute onset of head tremor may follow a paramedian pontomesencephalic infarct. An improvement of resting tremor in a patient with Parkinson disease after infarction in the territory of inferolateral and tuberothalamic arteries has been reported [59]. Acute onset of a “parkinsonian tremor” may be the earlier manifestation of stroke involving the medial tract of the substantia nigra [60]. Tremor and dystonia secondary to posterolateral thalamic infarctions were also documented in children [61]. Benedikit syndrome can induce controlateral involuntary movements, including intentional tremor, hemicorea, or hemiasthetosis, due to destruction of the red nucleus. Claude syndrome is another well known brain stem syndrome that may be associated with controlateral tremors. In vascular orthostatic tremor, a dopaminergic dysfunction may be involved [62]. Vascular tremor is particularly noncompliant to drug treatment. Rubral and palatal tremor may respond to clonazepam and sodium valproate [63]. Ondo *et al*., [64] illustrated a case about two patients with myoclonic tremor induced by parietal cortical lesions with tremulous finger movements induce by action and posture and had been successfully treated with both clonazepam and sodium valproate as first-line agents for orthostatic tremor [62]. Dystonic tremor is treated as dystonia [see above]. Conclusively, several reports have suggested that topiramate can play an important role in the treatment of several forms of tremor [65-68]. In particular, Connor [65] evaluated the effects of topiramate (400 mg/day or maximum tolerated dose) as monotherapy or as adjunctive treatment of essential tremor in a placebo-controlled, crossover study (n = 24) and demonstrated that topiramate was able to induce a significantly greater reduction from baseline based on normalized scores for a clinical rating of tremor location/severity, specific motor tasks/functional disabilities, and tremor-resultant functional disabilities. Several years later Connor *et al*., [66] combined the results of 3 randomized, double-blind, placebo-controlled, crossover trials which evaluated 62 patients randomized to a double-blind sequence of topiramate (400 mg/day or maximum tolerated dose) and then placebo (n = 30) or placebo and then topiramate (n = 32). Here, the authors documented that the total tremor score was significantly lower with topiramate (28.7 +/- 1.0) than with placebo (37.0 +/- 1.0), without any development of serious adverse events. In addition, Ondo *et al*., [67] in a multicenter, double-blinded, placebo-controlled, parallel-design trial, performed in 208 patients with moderate to severe essential tremor in the upper limbs were randomized to receive a 24 week treatment with either placebo or topiramate (400 mg/day) as monotherapy or as an adjunct to an antitremor medication. It documented that topiramate was effective in the treatment of moderate to severe essential tremor and that topiramate treatment helped to improve function and disabilities. Conclusevely, Siniscalchi *et al*., [68] illustrated the effects of both declorazepam (1 mg/day) and topiramate (200 mg/day) treatment on essential tremor in a 76-year-old man. 

## MYOCLONUS

Myoclonus is defined as sudden jerks typically lasting 10 to 50 milliseconds, with movements lasting rarely more than 100 milliseconds [69]. Myoclonus is usually a positive phenomenon, causing synchronized muscle contractions in single or multiple muscle groups. Myoclonus jerks can be irregular, rhythmic, or even oscillatory [69]. The dysfunction of inhibitory mechanisms may explain the neuronal hyperexcitability that underlies some forms of myoclonus. Therefore, myoclonus treatment aims to enhance the deficient GABAergic inhibitory neurotransmission [70]. Animal models of myoclonus have been generated by producing alterations of postsynaptic GABA-A and glycine receptors [71]. In general, valproate, levetiracetam and piracetam are effective in cortical myoclonus, but ineffective in other forms of myoclonus; while, clonazepam and sodium valproate are considered the first choice in the treatment for myoclonus [2]. Clonazepam is effective in all types of myoclonus and side-effects include sedation, vertigo, behavioural changes and tolerance and it is contraindicated in people with acute narrow-angle glaucoma and liver diseases [2,63]. Sodium valproate is effective in cortical and subcortical myoclonus. Side-effects include drowsiness, weight augumentation, tremor, nausea and alopecia. It is contraindicated in hepatic failure [63]. Moreover, on GABA-A receptor, levetiracetam is able to block the effects of the negative allosteric modulators [i.e. zinc] [72] and Schauer *et al*., [73] reported the efficacy of levetiracetam (1000 mg/day for 8 months) in a 16-year-old patient affected by disabling posthypoxic myoclonus.

Both piracetam and levetiracetam are very useful in myoclonus [74-76].

Genton and Gelise [74] illustrated a case series on three patients, one patient with severe post-anoxic myoclonus and the other two with Unverricht-Lundborg disease; they were treated with levetiracetam 4000 mg/day respectively for 16 days, 2 weeks and 14 weeks and received treatment with piracetam 27 and 36 mg/day as far as first and second patients were concerned, with a significant improvement of the symptoms. Moreover, in an open-labeled study, performed on sixty patients with disabling myoclonus, piracetam was reported to be effective, especially in cases with cortical origin [75].

In cortical myoclonus, piracetam or levetiracetam can be combined with sodium valproate and clonazepam. Piracetam is less sedating and may be as effective as levetiracetam [2] but both drugs should be used with caution in patients with renal failure.

Acetazolamide is a carbonic anhydrase inhibitor that has been reported to be useful in the treatment of myoclonus because it is able to enhance the GABA effect and the GABA levels in brain [77]. Primidone can be tried but it causes drowsiness, confusion and ataxia [21].

Treatment should begin with a single agent even if eventually several drugs in combination may be required.

## PARKINSONISM

Vascular parkinsonism has been associated with unilateral [78-80] or bilateral [81,82] basal ganglia infarcts in the striatum or lentiform nucleus. Parkinsonian symptoms may be caused by vascular lesions disrupting the interconnecting fibre tracts between basal ganglia, thalamus, and motor cortex leading to the disruption of sensory-motor integration [79-81] as well as descending reticular pathways to the major centres of the brain stem [80]. Two forms of vascular parkinsonism may be suggested: one with acute onset, related to basal ganglia infarcts, and another one with insidious progression, possibly associated with more scattered subcortical white matter ischaemia [81,82]. Previous studies have reported that the first distribution of vascular parkinsonism may be ipsilateral to the motor deficit or bilateral with predominance in the lower limb [81]. Supportive therapy physiotherapists and/or occupational therapists should be organized. Treatment of atherosclerosis i.e. anti-platelet agents, statins and anti-hypertensive drugs are also recommended.

Vascular parkinsonism has been considered a non responder [81,82] or poorly responsive to levodopa treatment [83-85]. However, a modest observational clinical trial performed in seventeen older patients with vascular parkinsonism, documented that L-dopa treatment (mean dose 450 mg/day, range 100–1000 mg/day) induced an excellent response in three patients, a good response in nine, and a moderate improvement in two patients during the first year, while three patients showed no response to L-dopa doses of 300–400 mg/day [14]. Reviewing literature data, Siniscalchi *et al*., [86] documented that in patients with Parkinson's disease, levodopa treatment can induce an increasement in serum homocysteine levels.

In agreement, Yong *et al*., [87] evaluated 17 patients with de novo Parkinson's disease, and documented a significant increase of homocysteine levels (13.3 vs 17.0 mg/dl; p < 0.001) after 3 months of levodopa/carbidopa treatment (450/75 mg/day), therefore suggesting that even if the short-term treatment with levodopa itself is not able to induce overall alteration of cerebral blood flow velocities and resistances, an increase in homocysteine serum levels may be able to induce cerebral vascular stiffness.

## CONCLUSION AND DIRECTIONS FOR FUTURE RESEARCH

Although rare, many different varieties of abnormal movements can be found after a stroke either acutely or as a delayed sequel. It can be hyperkinetic (most commonly hemichorea–hemiballismus) or hypokinetic (most commonly vascular parkinsonism). Most are caused by lesions in the basal ganglia or thalamus but can occur with strokes at many different locations in the motor circuit. Many are self-limiting but treatment may be required for symptom control. Previous clinical studies have reported that the post-stroke movement disorders had spontaneous regression generally within 2 weeks, only three patients [with dystonia or delayed complex movements] were resistant to medication and had persistant dyskinesias for *>*6 months [21]. An absence of essential tremor after stroke was observed in four patients and it may have been caused by the interruption of cerebellar loops during the stroke [88]*.* In other studies the post-stroke movements disorders worsened in time [2,7]. Alarcon *et al*. [9], showed that the spontaneous regression of post-stroke movement disorders in term of complete or partial resolution is dependent to the type of movement disorders. In fact, at 1-year follow-up, 10% of the patients with chorea had improved completely and 75% partially. Other patients had variable results; 28% of the patients with tremor acquired complete resolution (64% partial); 31% of the patients with dystonia had complete improvement (62% partial); in parkinsonism one out of six patients recovered spontaneously [9]. It has been documented that in most patients with hemiballisms, the prognosis is positive with complete resolution either with or without treatment [26]. However, the same authors reported that medical treatment is able to reduce or improve the movements in patients with major severe movement disorders [26]. The primary treatment of most hyperkinetic movement disorders [most commonly hemichorea–hemiballismus] is anti-dopaminergic therapy with typical and atypical neuroleptics and catecholamine-depleting agents [2,25], even if tetrabenazine and reserpine may also be used [2]. The modulation of GABAergic transmission could explain the neuronal hyper-excitability that underlies some forms of hyperkinetic movement disorders, therefore antiepileptic drugs are able to increase GABAergic neurotransmission (such as clonazepam, sodium Valproate) and can represent a useful therapeutic option in the management of post-stroke hyperkinetic movement disorders whenever the available treatments appear to be ineffective. 

No conclusive evidences regarding the efficacy of pharmacological treatment in patients with vascular tremor have been reported [14]. Moreover, there are no head-to-head trials comparing the different pharmacological treatments in post-stroke movement disorders therefore a direct comparison of both efficacy and tolerability of pharmacological treatments is impossible. The lack of previous research in such a matter represents a substantial challenge in the development of therapeutic recommendations for some post-stroke movement disorders. Research supporting the efficacy of antiepileptic drugs capable to enhance GABAergic neurotransmission in the management of post-stroke hyperkinetic movement disorders is still inadequate. Some post-stroke movement disorders have shown a partial benefit for pharmacological treatment or a drug resistance. In a study on patients with vascular hemiballismus treated with haloperidol ±diazepam, 56% of patients had complete recovery within 15 days, 37% had residual choreic movements and 7% had no response after 30 months [89]. Our systematic review shows that data regarding the pharmacological treatment of poststroke movements disorders are based only on case reports or modest clinical trials. 

Although functional recovery can occur spontaneously after ischemic stroke, often, it is partial and insufficient thus impossible to regain complete normal functioning [90]. In experimental models of ischemia, a spontaneous partial functional recovery from poststroke deficits is associated with the brain reorganization [91,92] and spontaneous poststroke changes in regional brain activity have been documented in these model systems *via *functional magnetic resonance imaging (fMRI) [93]. The changes in neuronal membrane excitability, the loss of perilesional GABAergic inhibition, the enhanced glutamatergic transmission and the post-ischemic synaptic plasticity contribute to brain reorganization post stroke suggest that pre-existing functionally silent synapses around the lesion are unmasked or disinhibited and that neuronal networks that are not normally involved, become progressively activated [94,95] Previous studies show that the bilateral activation of motor pathways and the recruitment of additional sensorimotor areas are associated with recovery from motor stroke due to striatocapsular infarction [96]. Similarly, the recovery from motor stroke is associated with individually different patterns of functional reorganization of the brain depending on both the site of the subcortical lesion and the somatotopic organization of the pyramidal tract [97].

An excitatory postsynaptic potential boost might deeply influence the plastic reorganization of cortical representational maps inducing a functional connection of previously non-interacting neurons or disconnecting formerly associated neurons. However, it is also possible to hypothesize that, in case of a subcortical infarct, the increased excitatory postsynaptic potential might strengthen the spared connections between cortical and subcortical neurons in order to allow preservation of a physiological output signal from the injured neural structure [95]. 

However, fMRI and magnetoencephalography (MEG) data confirm the association of brain remodelling and recovery from poststroke deficits in humans [92]. Brain reorganization appears to be the primary observable neuroanatomical phenomenon associated with functional recovery from stroke [98] thus therapies capable to enhance this limited plasticity may also produce an increasement in recoveries. Pharmacological agents, acknowledged to modulate practice-dependent plasticity in animal models of brain damage, have recently received increased interest for treatment of motor recovery after stroke [99,100].

Conclusevely, in the future, improved knowledge of the mechanisms underlying the effectiveness of some drugs in several post-stroke movement disorders should be provided in order to characterize the mechanisms of neuroplasticity that may be responsible of post-stroke recovery and may help to disclose new therapeutical approaches in patients with post-stroke movements disorders. 

## Figures and Tables

**Table 1.** Pharmacotherapy Options in Post-stroke Movement Disorders

Abnormal Movements	Pharmacotherapy Options:	Case Report	Clinical Trial	Review
*Hyperkinetic movements disorders* Hemichorea-Hemiballism	Typical neuroleptics—haloperidol, pimozide, perphenazine, fluphenazine Atypical neuroleptics —olanzapine, quetiapine, sulpiride, clozapine Tetrabenazine, reserpine Clonazepam Sodium valproate Levetiracetam Topiramate	*Refs* 36,44,45,46,47,48	*Refs* 34,35,41,43	*Refs* 2,33
Dystonia	Botulinum toxin Clonazepam, diazepam Baclofen Benzhexol, trihexyphenidyl Tetrabenazine		3,54	2,21,25,55
Tremor	Clonazepam Sodium valproate Propanololo	64,68	65,66,67	2
Myoclonus	Clonazepam, sodium valproate Levetiracetam, piracetam, primidone, acetazolamide	73,75	74	2,76,77
*Hypokietic movements disorders* Parkinsonism	partial benefit to dopaminergic therapy		81,84	
